# Digital twin technology in cardiovascular care: future perspectives for nursing practice, patient management, and clinical decision-making

**DOI:** 10.3389/fcvm.2026.1883153

**Published:** 2026-10-27

**Authors:** Naseema Shafqat, Veena Verma, Saman Naseema

**Affiliations:** 1 Department of Nursing College, All India Institute of Medical Sciences Bhopal, Bhopal, India; 2 Department of Nursing College, All India Institute of Medical Sciences Raipur, Raipur, India

**Keywords:** artificial intelligence, cardiovascular diseases, cardiovascular nursing, digital twin technology, precision cardiology, predictive analytics, remote patient monitoring, telecardiology

## Abstract

**Background and objective:**

Cardiovascular diseases remain the leading cause of mortality globally and continue to impose substantial clinical, economic, and organisational burdens on healthcare systems due to increasing disease complexity, recurrent hospitalisations, and the need for long-term monitoring and personalised care. Rapid advances in artificial intelligence, computational modelling, wearable biosensors, telehealth, and digital health technologies have accelerated the emergence of Digital Twin Technology (DTT) as a promising innovation in precision cardiovascular healthcare. Although DTT has gained growing attention in medicine and engineering, its implications for cardiovascular nursing practice, patient management, and clinical decision-making remain insufficiently synthesised. This scoping review aimed to explore the current applications of DTT in cardiovascular healthcare and examine its implications for cardiovascular nursing practice, patient management, clinical decision-making, and future healthcare delivery.

**Methods:**

Review was conducted using the Arksey and O'Malley methodological framework and reported according to the PRISMA-ScR guidelines. A comprehensive search of PubMed/MEDLINE, Scopus, Web of Science, CINAHL, Embase, IEEE Xplore, and Google Scholar was performed for studies published between 2020 and 2026. Eligible studies included literature related to digital twins, computational cardiovascular modelling, artificial intelligence-assisted cardiovascular care, wearable technologies, remote monitoring systems, and nursing implications in cardiovascular healthcare. Data were charted and synthesised narratively according to predefined research questions and thematic areas

**Results:**

Thirty studies met the inclusion criteria. The findings demonstrated that DTT is increasingly applied in heart failure monitoring, arrhythmia prediction, telecardiology, smart ICU systems, virtual cardiac modelling, personalised rehabilitation, and population-level cardiovascular risk prediction. Artificial intelligence, machine learning, wearable biosensors, cloud computing, electronic health records, and predictive analytics were the most commonly reported technological components. The review further identified important implications for cardiovascular nursing, including transition from reactive to predictive care, expansion of telehealth nursing, enhanced data-driven clinical decision-making, and the growing need for digital competency. However, ethical concerns, cybersecurity risks, interoperability challenges,infrastructure costs, workforce preparedness, and limited nursing-focused evidence remain significant barriers.

**Conclusion:**

DTT has the potential to transform cardiovascular healthcare through predictive, personalised, and continuously adaptive models of care. Strengthening nursing leadership, digital competency and interdisciplinary collaboration is essential for integration of DTT into future cardiovascular practice.

## 1 Introduction

Cardiovascular diseases (CVDs) remain the leading cause of mortality and long-term disability worldwide, accounting for a substantial proportion of global healthcare burden despite significant progress in prevention and treatment (1, 2). Conditions such as coronary artery disease, heart failure, hypertension, arrhythmias, and stroke continue to contribute to repeated hospitalisation, reduced quality of life, impaired functional capacity, and premature mortality across both developed and developing countries (3, 4). The burden of cardiovascular disease has further increased due to population ageing, sedentary lifestyles, obesity, diabetes mellitus, tobacco consumption, unhealthy dietary behaviours, and rising psychosocial stress (2, 5). Consequently, healthcare systems are facing increasing pressure to deliver more efficient, personalised, and sustainable cardiovascular care while simultaneously improving patient outcomes and continuity of care.

Over the past two decades, major advances in diagnostic imaging, interventional cardiology, pharmacological therapies, and chronic disease management have considerably improved survival among patients with cardiovascular diseases (5). However, these advancements have also increased the complexity of cardiovascular care by creating greater demands for continuous physiological monitoring, rapid clinical decision-making, multidisciplinary collaboration, and long-term patient management (4). Modern cardiovascular healthcare is therefore progressively shifting from episodic hospital-based treatment towards preventive, predictive, and patient-centred models of care that support long-term disease surveillance and early intervention (3).

Simultaneously, healthcare systems worldwide are undergoing rapid digital transformation through the integration of electronic health records, wearable biosensors, telemedicine, mobile health applications, cloud computing, and the Internet of Medical Things (IoMT) (6, 7). These innovations have significantly improved healthcare communication, accessibility of patient information, remote monitoring, and patient participation in self-management (8). In cardiovascular healthcare, digital technologies are increasingly enabling healthcare professionals to identify early clinical deterioration, initiate timely interventions, and improve continuity of care through remote and technology-assisted monitoring systems (8, 9).

Despite these developments, traditional approaches to cardiovascular risk assessment and disease management continue to rely largely on population-based models that may not adequately capture the complexity and variability of individual patient trajectories (10). Patients with similar diagnoses frequently demonstrate markedly different disease progression, treatment responses, and clinical outcomes due to variations in genetics, environmental exposures, comorbidities, lifestyle behaviours, and psychosocial factors (11). This growing recognition of interindividual variability has intensified interest in predictive and precision-based approaches to cardiovascular care.

One of the most promising innovations emerging from this digital healthcare revolution is Digital Twin Technology (DTT). The concept of the digital twin originated in the aerospace and engineering sectors, where virtual replicas of physical systems were developed to simulate performance, predict failures, and optimise system functioning (12). In healthcare, a digital twin refers to a dynamic virtual representation of an individual patient or biological system that continuously updates through integration of real-time physiological and clinical data, including imaging, genomics, wearable sensor outputs, laboratory findings, and electronic health records (13, 14). By combining computational modelling, artificial intelligence, machine learning, and predictive analytics, digital twins can simulate disease progression, estimate clinical outcomes, evaluate treatment responses, and support personalised clinical decision-making (15, 16).

Cardiovascular care is particularly well suited for digital twin integration because of the highly dynamic and data-intensive nature of cardiac monitoring and disease management (15). Cardiovascular diseases generate large volumes of measurable physiological data through electrocardiography, echocardiography, haemodynamic monitoring, imaging systems, implantable cardiac devices, and wearable technologies (9). This continuous flow of patient-specific information provides an ideal foundation for developing personalised computational models capable of predicting disease progression, identifying early deterioration, and evaluating therapeutic responses before clinical events occur (15). Furthermore, digital twin systems may support precision cardiovascular care by enabling virtual simulation of interventions and treatment strategies before implementation in real clinical settings (17).

For nursing practice, DTT may offer opportunities to strengthen patient monitoring, support patient education, facilitate proactive disease management, and assist clinical decision-making (10). Though, these applications remain emerging, as relatively few studies have directly evaluated DTT within cardiovascular nursing practice. As healthcare systems increasingly adopt intelligent digital technologies, nurses are expected to develop competencies in digital literacy, nursing informatics, interpretation of predictive analytics, and technology-assisted clinical care (16). Cardiovascular nurses may be well positioned to contribute to patient engagement, coordination of technology-enabled care, telemonitoring, and patient-centred implementation of DTT. However, these roles require further empirical evaluation and should not yet be considered established components of digital twin-enabled nursing practice.

Nevertheless, despite growing interest in DTT within medicine, engineering, and computational sciences, relatively limited literature has specifically examined its implications for cardiovascular nursing practice. Previous reviews of cardiovascular digital twins, precision cardiology, and AI-enabled cardiovascular care have predominantly focused on technological development, computational modelling, clinical applications, and precision medicine, with comparatively limited attention to nursing integration, patient-centred implementation, and cardiovascular nursing roles**.** The present scoping review therefore extends the existing literature by bringing together evidence on nursing roles, workflow integration, patient monitoring, care coordination, nursing competencies, and implementation challenges, while also considering ethical and regulatory issues, digital equity, and future research priorities. Successful integration of DTT requires robust ethical governance, workforce preparedness, digital competency development, interdisciplinary collaboration, and supportive healthcare policies to ensure safe and equitable implementation in clinical practice (18, 19). The overall architecture and functional components of a cardiovascular digital twin system are illustrated in Figure 1.

**Figure 1:**
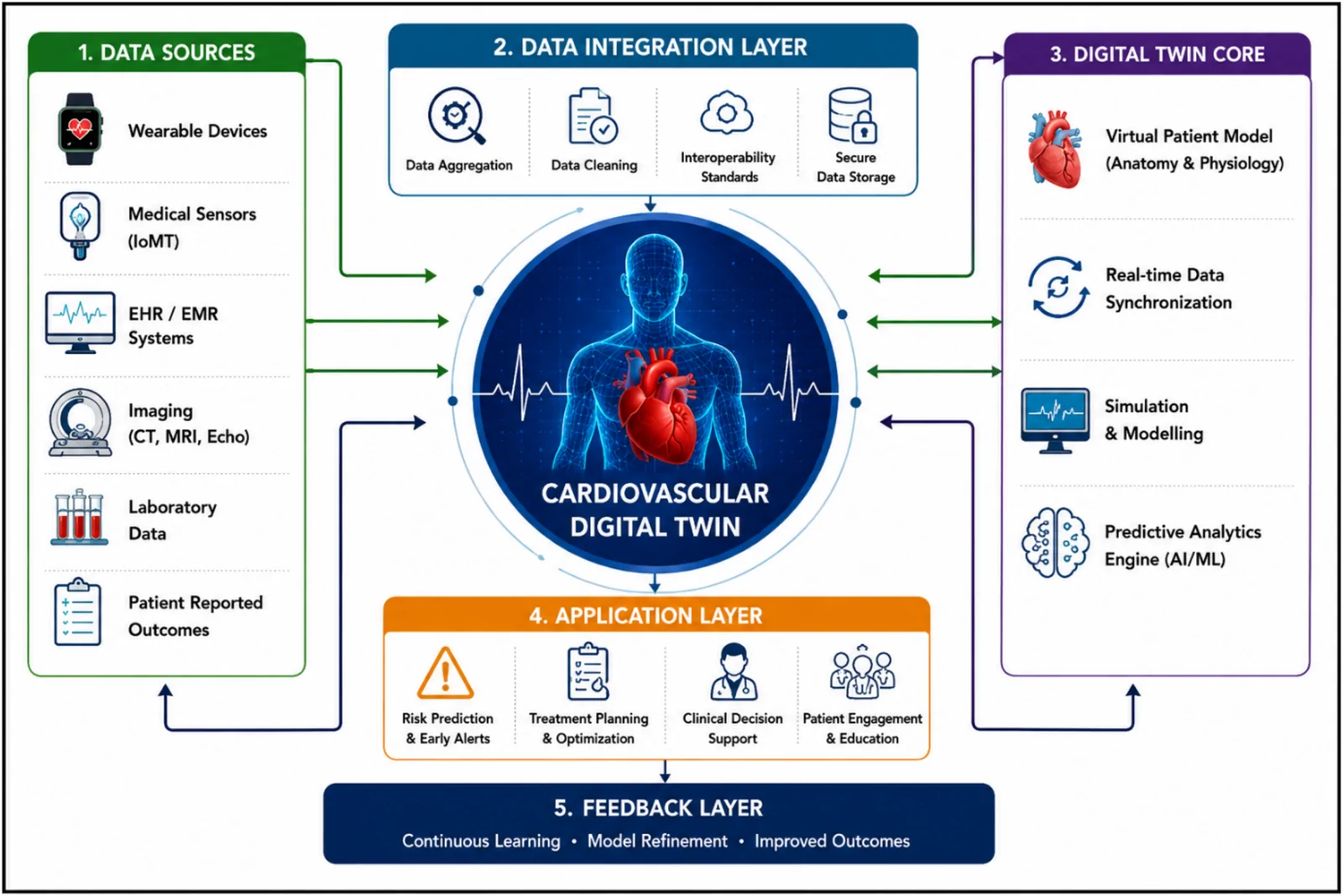
Architecture of a cardiovascular digital twin system (conceptual representation of data integration, digital twin modelling, clinical applications, and continuous feedback in cardiovascular care). Author-developed conceptual figure based on the synthesis of the reviewed literature; not a validated clinical model*.*

Therefore, this scoping review aims to synthesise the current evidence regarding the applications of Digital Twin Technology in cardiovascular care, with particular emphasis on cardiovascular nursing practice, patient management, and clinical decision-making. In addition, the review seeks to identify current research gaps, implementation challenges, and future directions for nursing education, policy development, and clinical practice within this rapidly evolving field of cardiovascular healthcare.

## 2 Materials and methods

This scoping review was conducted using the methodological framework developed by Arksey and O'Malley (20), further refined by Levac et al. (21), and reported in accordance with the Preferred Reporting Items for Systematic Reviews and Meta-Analyses Extension for Scoping Reviews (PRISMA-ScR) guidelines proposed by Tricco et al. (22). The review specifically focused on the applications of digital twin systems in cardiovascular healthcare and their implications for cardiovascular nursing practice, patient management, and clinical decision-making. The review process followed six methodological stages: (1) identifying the research questions; (2) identifying relevant studies through a comprehensive search strategy; (3) study selection; (4) charting the data; (5) collating, summarising, and reporting the findings; and (6) consultation exercise (20–22).

### 2.1 Identifying the research questions

The review was guided by the following research questions:

1. What are the current applications of Digital Twin Technology in cardiovascular care and patient management?
2. How can Digital Twin Technology support cardiovascular nursing practice, including patient monitoring, clinical decision-making, and remote care?
3. What are the potential benefits and challenges associated with the implementation of Digital Twin Technology in cardiovascular healthcare?
4. What gaps exist in the current literature regarding the integration of Digital Twin Technology into cardiovascular nursing practice and future healthcare systems?

These research questions were developed to explore the current scope of evidence related to digital twin applications in cardiovascular healthcare and to identify implications for future nursing practice, education, and research.

### 2.2 Identifying relevant studies—search strategy

A comprehensive and systematic literature search was conducted across multiple electronic databases, including PubMed/MEDLINE, Scopus, Web of Science, CINAHL, Embase, IEEE Xplore, and Google Scholar. Additional manual searches of reference lists from relevant studies were also performed to identify potentially eligible articles that may not have been retrieved through the primary database search. The search strategy was developed using a combination of Medical Subject Headings (MeSH), controlled vocabulary, Boolean operators, truncation techniques, and free-text keywords. The primary search terms included “Digital Twin”, “Digital Twin Technology”, “Virtual Patient Model”, “Artificial Intelligence”, “Predictive Analytics”, “Cardiovascular Care”, “Cardiology”, “Heart Disease”, “Cardiovascular Nursing”, “Nursing Practice”, “Clinical Decision-Making”, “Remote Monitoring”, “Precision Medicine”, and “Digital Health”. Boolean operators such as “AND” and “OR” were used to combine search terms and improve search sensitivity. Only studies published in English between 2020 and 2026 were considered eligible for inclusion in order to capture recent evidence related to emerging digital healthcare technologies and cardiovascular digital twin applications.

### 2.3 Study selection

Predefined eligibility criteria were developed and applied during the study selection process, as presented in Table 1. Study screening was conducted independently by two reviewers through assessment of titles and abstracts identified during the database search. Articles considered potentially relevant were selected for further review. In situations where the abstract did not provide sufficient information to determine eligibility, the full-text article was reviewed before making a final inclusion decision. Any disagreements between the two reviewers regarding study selection were resolved through discussion and mutual consensus.

**Table 1:** Inclusion and exclusion criteria.

Criteria	Inclusion criteria	Exclusion criteria
Publication date	Published between 2020 and 2026	Published before 2020
Language	Articles published in English	Articles published in languages other than English
Study design	Original research studies including quantitative studies, qualitative studies, mixed-method studies, observational studies, cohort studies, pilot studies, feasibility studies, case studies, scoping reviews, and systematic reviews related to Digital Twin Technology in cardiovascular care	Editorials, commentaries, conference abstracts without full text, letters to the editor, opinion papers, dissertations, non-scientific reports, and duplicate publications
Participants/Population	Adult patients with cardiovascular diseases, healthcare professionals, nurses, multidisciplinary cardiovascular care teams, and healthcare systems involved in cardiovascular care	Studies unrelated to cardiovascular healthcare or studies exclusively involving non-healthcare industrial applications
Intervention/Concept	Studies focusing on Digital Twin Technology, artificial intelligence-supported cardiovascular systems, predictive cardiac modelling, virtual patient models, remote cardiac monitoring, telecardiology, wearable cardiovascular technologies, and intelligent cardiovascular healthcare systems	Studies focusing solely on conventional digital health technologies without any relevance to digital twins, predictive modelling, or cardiovascular applications
Context	Cardiovascular care settings including hospitals, cardiac intensive care units, outpatient departments, rehabilitation centres, community healthcare, and home-based cardiac monitoring	Studies unrelated to cardiovascular care settings
Outcomes	Studies reporting applications, effectiveness, clinical implications, nursing implications, predictive monitoring, patient outcomes, clinical decision-making, ethical considerations, implementation challenges, or future perspectives related to digital twin technology	Studies without relevance to cardiovascular nursing practice, patient management, or clinical applications
Accessibility	Full-text articles available through electronic databases	Articles with unavailable full text or incomplete data

### 2.4 Methodological quality assessment

Formal methodological quality or risk-of-bias assessment was not undertaken because this scoping review included heterogeneous publication types, including conceptual, modelling, review, and other evidence-based studies for which a single standardized appraisal tool was not appropriate. Consistent with the exploratory purpose of a scoping review, the included evidence was mapped and synthesised according to study design, application, nursing relevance, and reported findings.

### 2.5 Charting the data

A standardised data-charting form was developed to systematically extract and organise information from the included studies. Extracted data included author details, year of publication, country of study, study design, type of digital twin application, cardiovascular context, nursing relevance, technological components, key findings, implementation challenges, and future implications for practice and research. Data extraction was conducted independently by two reviewers and cross-checked to ensure consistency, completeness, and accuracy of the extracted information.

### 2.6 Collating, summarising, and reporting the results

The extracted data were collated, summarised, and synthesised using a narrative descriptive approach. The findings from the included studies were organised according to thematic areas related to the application of Digital Twin Technology in cardiovascular care and its implications for nursing practice, patient management, and clinical decision-making.

For Research Question 1, the included studies were analysed to identify the major domains of digital twin applications in cardiovascular healthcare. The findings were summarised according to common characteristics such as cardiovascular conditions addressed, purpose of digital twin implementation, technological components utilised, artificial intelligence integration, predictive modelling approaches, and clinical settings. The applications were further categorised into areas including early risk prediction, remote cardiac monitoring, personalised treatment planning, rehabilitation support, surgical simulation, and population health management.

For Research Question 2, studies were analysed to identify nursing-related implications of digital twin integration. The findings were summarised according to themes including nursing assessment, predictive monitoring, telehealth integration, virtual nursing models, patient education, continuity of care, and digital competency requirements. Particular emphasis was placed on the evolving role of cardiovascular nurses in technology-assisted healthcare environments.

For Research Question 3, the extracted findings were synthesised according to reported clinical benefits, organisational outcomes, ethical concerns, and implementation barriers. The potential benefits were grouped under themes such as improved patient safety, early detection of deterioration, enhanced clinical decision-making, reduced hospitalisation, and personalised care. Reported challenges included technological limitations, interoperability issues, infrastructure costs, cybersecurity concerns, ethical considerations, workforce preparedness, and barriers to implementation in low- and middle-income countries.

For Research Question 4, the included studies were reviewed to identify existing gaps in the literature and areas requiring future investigation. Research gaps were summarised according to themes including limited nursing-focused research, lack of longitudinal clinical studies, inadequate cost-effectiveness evaluations, digital competency training needs, ethical framework development, patient acceptance studies, and applicability in resource-limited settings.

The findings are presented descriptively using tables and narrative summaries to provide a comprehensive overview of current evidence related to digital twin technology in cardiovascular healthcare. Due to heterogeneity in study designs, technological approaches, and reported outcomes, the results were synthesised qualitatively rather than through meta-analysis.

### 2.7 Consultation exercise

To enhance the practical relevance and applicability of the review findings, a consultation exercise was conducted with experts from cardiovascular nursing, digital health, and cardiovascular medicine. The consultation process provided additional insights into the clinical applicability, implementation challenges, future opportunities, and nursing implications of Digital Twin Technology in cardiovascular care. Feedback obtained during the consultation exercise helped validate the thematic findings, identify additional research gaps, and strengthen interpretation of the results related to nursing practice, digital competency, and future healthcare integration.

### 2.8 Ethical considerations

As this scoping review involved secondary analysis of previously published literature, ethical approval was not required. However, the review was conducted according to principles of transparency, scientific integrity, and accurate reporting throughout all stages of the review process.

## 3 Results

### 3.1 Study selection process

A total of 742 studies were identified through database searching across PubMed/MEDLINE, Scopus, Web of Science, CINAHL, Embase, IEEE Xplore, and Google Scholar, with an additional 18 studies identified through manual searching of reference lists and relevant review articles. After removal of 126 duplicates, 634 studies underwent title and abstract screening, of which 521 were excluded for not meeting the inclusion criteria. Full-text assessment was conducted for 113 studies, and 83 studies were further excluded because they lacked specific relevance to cardiovascular digital twin systems, nursing implications, or clinical applications. Ultimately, 30 studies published between 2020 and 2026 were included in the scoping review. The included studies primarily focused on precision cardiology, computational cardiac modelling, artificial intelligence-assisted monitoring, remote cardiovascular care, wearable technologies, predictive analytics, telecardiology, and digital healthcare ecosystems, with several studies also addressing implications for cardiovascular nursing practice and clinical decision-making (See Figure 2 for study selection process).

**Figure 2:**
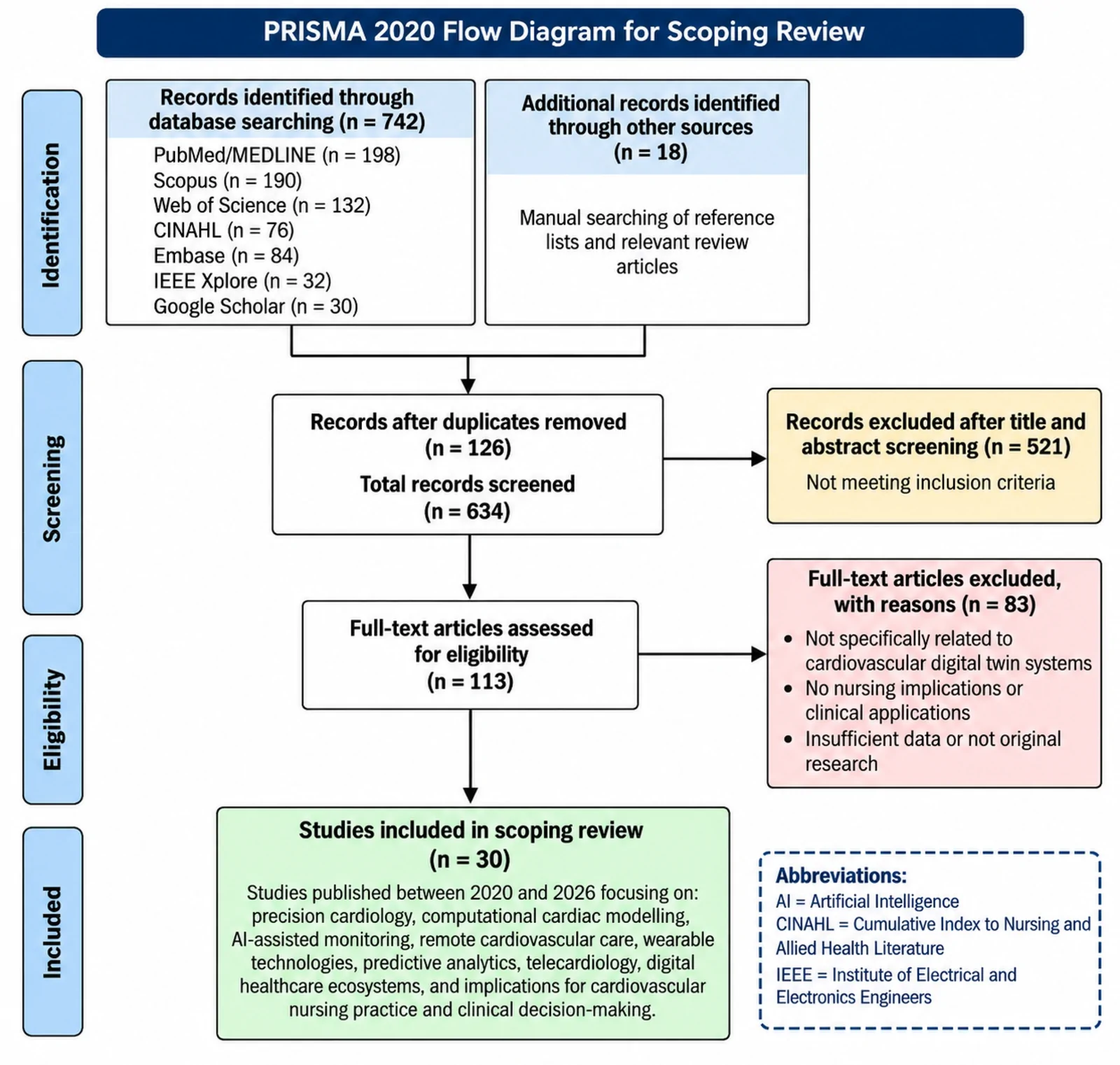
PRISMA-ScR flow diagram of study selection.

### 3.2 Characteristics of included studies

The 30 included studies were analysed according to year of publication, country and healthcare setting, study design, cardiovascular conditions addressed, digital twin technologies utilised, and nursing-related focus areas (11, 13, 15). Most studies were published between 2020 and 2024, reflecting the rapidly emerging nature of Digital Twin Technology (DTT) within cardiovascular healthcare (10, 13, 15). Foundational conceptual and precision cardiology studies published in 2020 primarily focused on computational cardiovascular modelling, personalised medicine, and patient-specific virtual heart systems (12–15). A substantial increase in publications was observed from 2021 onwards, particularly in areas related to artificial intelligence-assisted cardiovascular monitoring, wearable technologies, telecardiology, predictive analytics, and smart healthcare ecosystems (23–27). Recent studies further demonstrated growing interest in generative artificial intelligence, precision cardiovascular medicine, and integrated digital healthcare ecosystems (10, 11, 28, 29).

Majority of studies originated from technologically advanced healthcare systems in North America and Europe (11, 15, 26). The United States contributed the largest number of studies, followed by the United Kingdom, Sweden, Italy, Japan, Australia, Germany, Canada, Spain, South Korea, and multinational European collaborations (13, 23, 30–32). The studies were mainly conducted within tertiary hospitals, academic medical centres, cardiovascular research institutes, biomedical engineering laboratories, and interdisciplinary digital health research settings (9, 15, 33). Several studies additionally examined digital twin applications in telehealth services, remote cardiac monitoring systems, home-based cardiovascular care, and virtual healthcare ecosystems (24, 25, 27). Limited evidence was identified from low- and middle-income countries, indicating inequalities in digital infrastructure, technological accessibility, and implementation capacity (10, 29).

Narrative reviews, conceptual reviews, interdisciplinary reviews, and computational modelling studies represented the largest proportion of included studies (12–15). These studies mainly explored conceptual frameworks, technological integration, implementation opportunities, and future applications of DTT in cardiovascular care (10, 11, 28). Several studies involved computational cardiac modelling, electrophysiological simulation, predictive analytics, artificial intelligence-assisted monitoring systems, wearable biosensor integration, and telecardiology technologies (30, 33–35). Only a limited number of studies evaluated real-world clinical implementation or longitudinal cardiovascular outcomes associated with digital twin systems (23, 26). Nursing-focused studies were comparatively limited and mainly addressed telehealth integration, nursing informatics, digital competency, and predictive monitoring systems (24, 27, 31).

Heart failure was the most frequently discussed cardiovascular condition because of its chronic disease trajectory, recurrent hospitalisation, and need for continuous physiological monitoring (23, 36, 45). Other commonly addressed cardiovascular conditions included arrhythmias, ventricular tachycardia, atrial fibrillation, coronary artery disease, hypertension, hypertrophic cardiomyopathy, myocardial infarction, and structural heart disease (8, 30, 33, 34, 37). Several studies also explored haemodynamic instability, electrophysiological simulation, sudden cardiac event prediction, cardiac imaging analysis, and personalised intervention planning (9, 35). In addition, some studies focused on preventive cardiology, cardiovascular risk stratification, and population-level cardiovascular disease prediction models (29, 38).

Artificial intelligence and machine learning were the most frequently reported technological components supporting cardiovascular digital twin systems (10, 11, 28). These technologies were mainly utilised for predictive analytics, risk stratification, computational cardiac modelling, and clinical decision-support systems (8, 39). Wearable biosensors, remote cardiac monitoring devices, mobile health applications, Internet of Medical Things (IoMT) systems, cloud computing platforms, and electronic health record integration were also commonly identified (23–25, 40). Several studies additionally explored electrophysiological simulations, virtual physiological models, finite element modelling, computational haemodynamic modelling, and precision medicine frameworks as essential components of future cardiovascular digital twin ecosystems (9, 30, 33). (Table 2).

**Table 2:** Characteristics of included studies on digital twin technology in cardiovascular care (2020–2026).

S.No	Author(s)	Year	Country	Study design	Type of digital twin application	Cardiovascular context	Nursing relevance	Technological components	Key findings	Implementation challenges	Future implications for practice and research
1.	Corral-Acero et al. (15)	2020	Europe	Scientific review	Digital twin for precision cardiology	Heart failure, arrhythmias, cardiac modelling	Limited direct nursing focus	AI, computational modelling, ECG integration, imaging	Digital twins improved personalised cardiovascular treatment and predictive cardiology	Limited clinical validation and real-world implementation	Precision medicine and virtual patient simulation
2.	Bruynseels et al. (14)	2018	Belgium	Conceptual review	Patient digital twin systems	Personalised cardiovascular medicine	Minimal nursing focus	Big data, AI, machine learning	Potential for highly individualised cardiovascular care pathways	Ethical and legal concerns	Development of patient-centred digital healthcare
3.	Björnsson et al. (13)	2020	Sweden	Conceptual paper	Patient-specific digital twins for personalised medicine	Precision medicine and chronic disease management	Moderate nursing relevance	Multi-omics, AI, computational modelling, EHRs, wearables	Strong framework for predictive and personalised care	Data integration complexity	Future personalised cardiovascular digital ecosystems
4.	Fuller et al. (12)	2020	Multi-national	Systematic review	Digital twin conceptual healthcare framework	General healthcare and cardiovascular applications	Indirect nursing relevance	IoT, AI, cloud systems, data analytics	Established foundational digital twin healthcare architecture	Interoperability and infrastructure issues	Expansion of digital twin-enabled healthcare systems
5.	Niederer et al. (9)	2019	United Kingdom	Computational cardiology review	Mechanistic cardiac modelling	Electrophysiology and arrhythmia modelling	Supports advanced cardiac monitoring	MRI-guided modelling, computational simulation	Improved prediction of cardiac electrical abnormalities	Complexity of model development	Enhanced predictive cardiac diagnostics
6.	Trayanova et al. (33)	2020	USA	Precision cardiology modelling review	Personalised virtual heart modelling	Ventricular arrhythmias and ablation planning	Moderate nursing relevance	AI-guided simulations, electrophysiological modelling	Improved arrhythmia prediction and procedural planning	High computational demands	Personalised electrophysiology and ablation planning
7.	Gilbert et al. (39)	2020	United Kingdom	Review article	Statistical cardiovascular digital twins	Hypertrophic cardiomyopathy and ECG phenotyping	Indirect nursing relevance	Machine learning, ECG analytics	Integration of AI and predictive cardiac analytics	Data interpretation limitations	Predictive cardiovascular diagnostics
8.	Piersanti et al. (36)	2020	Italy	Clinical modelling framework	Hybrid statistical–mechanistic digital twin	Heart failure and cardiac imaging	Indirect nursing relevance	Computational imaging, AI models	Improved hybrid precision cardiology modelling	Clinical translation challenges	AI-assisted precision cardiac care
9.	Vigmond et al. (35)	2020	Canada	Electrophysiological modelling study	Virtual electrophysiological heart twin	Ventricular tachycardia	Limited nursing relevance	Electrophysiological simulations	Improved arrhythmia prediction	Technical simulation complexity	Better procedural planning
10.	Narigina et al. (41)	2025	Latvia	Review article	Mechanistic and statistical hybrid twin systems	General cardiovascular disease modelling	Moderate nursing relevance	AI, computational physiology	Combined modelling enhanced predictive accuracy	Computational integration challenges	Broader clinical adoption of digital twins
11.	Kamel Boulos et al. (25)	2021	United Kingdom	Narrative review	AI-assisted healthcare digital twins	Remote cardiovascular monitoring	Supports telehealth nursing models	IoMT, wearable devices, AI, telehealth	Improved continuity of cardiovascular care	Cybersecurity and data governance concerns	Expansion of home-based cardiac care
12.	Krittanawong et al. (8)	2021	USA	Narrative review	AI and precision cardiology digital twins	Coronary artery disease and arrhythmias	Supports evidence-based cardiovascular nursing	Machine learning, predictive analytics	Improved risk stratification and decision-making	Data integration limitations	Expansion of predictive cardiology
13.	Gerach et al. (34)	2021	Germany	Computational study	Left atrial scar digital twin	Atrial fibrillation	Supports cardiac monitoring	MRI imaging, electrophysiological simulation	Improved prediction of atrial fibrillation recurrence	Complex imaging integration	Personalised electrophysiological interventions
14.	Kagiyama et al. (31)	2019	Japan	Practical review	AI-assisted cardiology	Heart disease diagnosis and risk prediction	Highlights need for AI competency among nurses	AI algorithms, predictive analytics	AI improved cardiovascular diagnostic precision	Ethical and interpretational concerns	AI-supported clinical decision-making
15.	Kim et al. (40)	2021	South Korea	Wearable technology study	Wearable biosensor monitoring	Cardiovascular disease prevention	Supports patient-centred nursing surveillance	Wearable sensors, physiological tracking	Improved patient engagement and prevention	Data accuracy variability	Long-term preventive monitoring
16.	Jones et al. (24)	2021	United Kingdom	Review article	Digital health intervention systems	Telecardiology and remote care	Supports virtual nursing and telehealth	Telemedicine platforms, mobile apps	Improved accessibility to cardiovascular services	Infrastructure limitations	Broader telehealth implementation
17.	Coorey et al. (23)	2022	Australia	Review article	AI-supported remote cardiac monitoring	Heart failure management	Supports nurse-led remote monitoring	Wearable biosensors, predictive analytics	Early identification of deterioration reduced admissions	Patient adherence and technological acceptance	Broader telecardiology integration
18.	Musleh et al. (26)	2023	USA	Review article	Digital twin-enabled smart ICU systems	Critical cardiovascular care	Enhances nursing surveillance and prioritisation	Smart ICU systems, AI alerts, cloud computing	Improved detection of haemodynamic instability	Infrastructure cost and workflow adaptation	Intelligent ICUs and predictive critical care
19.	Saddik et al. (27)	2023	UAE	Review article	Telehealth-integrated digital twins	Chronic cardiovascular disease management	Strong relevance to virtual nursing	Telehealth systems, wearable monitoring	Improved continuity of care and remote follow-up	Limited digital literacy	Expansion of nurse-led virtual care
20.	Shade et al. (38)	2023	USA	Review article	Federated learning digital twin	Population cardiovascular modelling	Supports population health nursing	Federated AI learning, EHR integration	Improved large-scale cardiovascular prediction	Data-sharing restrictions	Predictive epidemiology and public health planning
21.	Kirkiles et al. (42)	2023	USA	Modelling studies	Digital twin in cardiomyopathy	Arrhythmogenic and hypertrophic cardiomyopathy	Supports predictive monitoring	MRI, AI-based modelling	Improved disease progression prediction	Computational complexity and limited validation	Personalised electrophysiological care
22.	Teodoru (43)	2026	Romania	Review article	AI-based diagnostic enhancement	Early cardiovascular diagnosis	Supports rapid clinical assessment	AI-assisted diagnostics, imaging analytics	Improved early disease identification	Regulatory and reliability concerns	Smart diagnostic healthcare systems
23.	Shao X et al. (44)	2025	Multinational	Review article	Digital therapeutics in cardiovascular disease	Chronic cardiovascular disease management	Supports patient coaching and rehabilitation	Digital therapeutics, mobile platforms	Improved long-term disease management	Regulatory and adoption barriers	Future personalised therapeutic ecosystems
24.	Thangaraj et al. (10)	2024	Sweden/USA/Canada	State-of-the-art review	Cardiovascular digital twin ecosystem	Precision cardiology and predictive cardiovascular care	Highlights need for digital competency and predictive nursing care	AI, generative AI, EHRs, wearable sensors	Improved personalised cardiovascular management	Ethical concerns and interoperability issues	Integration into mainstream cardiovascular care
25.	Nooruddin et al. (28)	2024	USA	Interdisciplinary review	Generative AI-supported cardiovascular digital twins	Smart cardiovascular healthcare systems	Discusses evolving nursing roles	Generative AI, EHRs, IoMT	Improved predictive modelling and workflow efficiency	Ethical AI governance concerns	Autonomous AI-assisted cardiovascular care
26.	Camps et al. (30)	2024	Spain	Computational modelling study	Purkinje network digital twin	Hypertrophic cardiomyopathy	Indirect nursing relevance	ECG/CMR modelling, finite element modelling	Enhanced simulation of arrhythmogenic pathways	High computational requirements	Improved patient-specific arrhythmia management
27.	Li et al. (37)	2024	United Kingdom	Experimental modelling study	MRI scar-based digital twin	Myocardial infarction	Supports rehabilitation monitoring	ECG/CMR integration, AI modelling	Accurate prediction of myocardial scar behaviour	Validation challenges	Precision rehabilitation planning
28.	Tamura et al. (29)	2024	Japan	Review article	Digitalomics and cardiovascular digital ecosystem	Precision cardiology and digital healthcare	Highlights future need for digitally competent nurses	AI, wearables, telehealth, EHRs	Digital technologies support predictive cardiovascular care	Digital divide and privacy concerns	Future digital cardiovascular ecosystems
29.	Nazir et al. (32)	2025	Multinational	Clinical review	Wearable cardiovascular monitoring technologies	Cardiovascular disease prevention	Strong relevance to nurse-led monitoring	Wearable ECG devices, mobile monitoring	Improved self-management and continuous monitoring	Digital literacy and adherence barriers	Wider adoption of home-based monitoring
30.	Antoun et al. (11)	2026	United Kingdom	Narrative review	Digital twin-enabled personalised cardiovascular medicine	Precision cardiology and prevention	Highlights future need for digitally skilled nurses	AI, genomics, machine learning, EHRs	Precision cardiology may transform cardiovascular prevention	Regulatory uncertainty and equity concerns	Shift towards proactive cardiovascular care

### 3.3 Nursing-related focus of included studies

Only a limited proportion of studies specifically addressed the implications of DTT for cardiovascular nursing practice (10, 24, 27). Most nursing-related studies broadly focused on digital transformation in healthcare, artificial intelligence in nursing, telehealth integration, nursing informatics, and digital competency development (28, 29, 31). The nursing-focused literature mainly discussed predictive patient monitoring, remote care coordination, virtual nursing models, patient education, interpretation of digital health data, and technology-assisted clinical decision-making (23, 24, 26). Very few studies directly explored nurse-led digital twin interventions, cardiovascular nursing workflow integration, or nursing-specific implementation frameworks (10, 27). Similarly, limited evidence was identified regarding nursing education preparation, ethical concerns from nursing perspectives, and digital twin competency frameworks for cardiovascular nurses (11, 29). These findings highlight a substantial gap in the literature and emphasise the need for future nursing-led research related to Digital Twin Technology in cardiovascular care (10, 28).

### 3.4 Applications of digital twin technology in cardiovascular care and patient management

The reviewed studies demonstrated that Digital Twin Technology (DTT) is increasingly being applied across multiple domains of cardiovascular healthcare, particularly in predictive monitoring, personalised treatment planning, remote patient management, precision cardiology, and intelligent critical care systems (10, 11, 15). The literature consistently described DTT as an advanced computational approach that creates dynamic virtual representations of patients by integrating real-time physiological data, artificial intelligence (AI), machine learning, imaging systems, wearable technologies, and electronic health records (12, 13, 28). The included studies suggested that cardiovascular care is particularly suitable for DTT integration because cardiac diseases require continuous monitoring, long-term management, and timely clinical decision-making (8, 23). The workflow of digital twin-enabled cardiovascular care is summarised in Figure 3.

**Figure 3:**
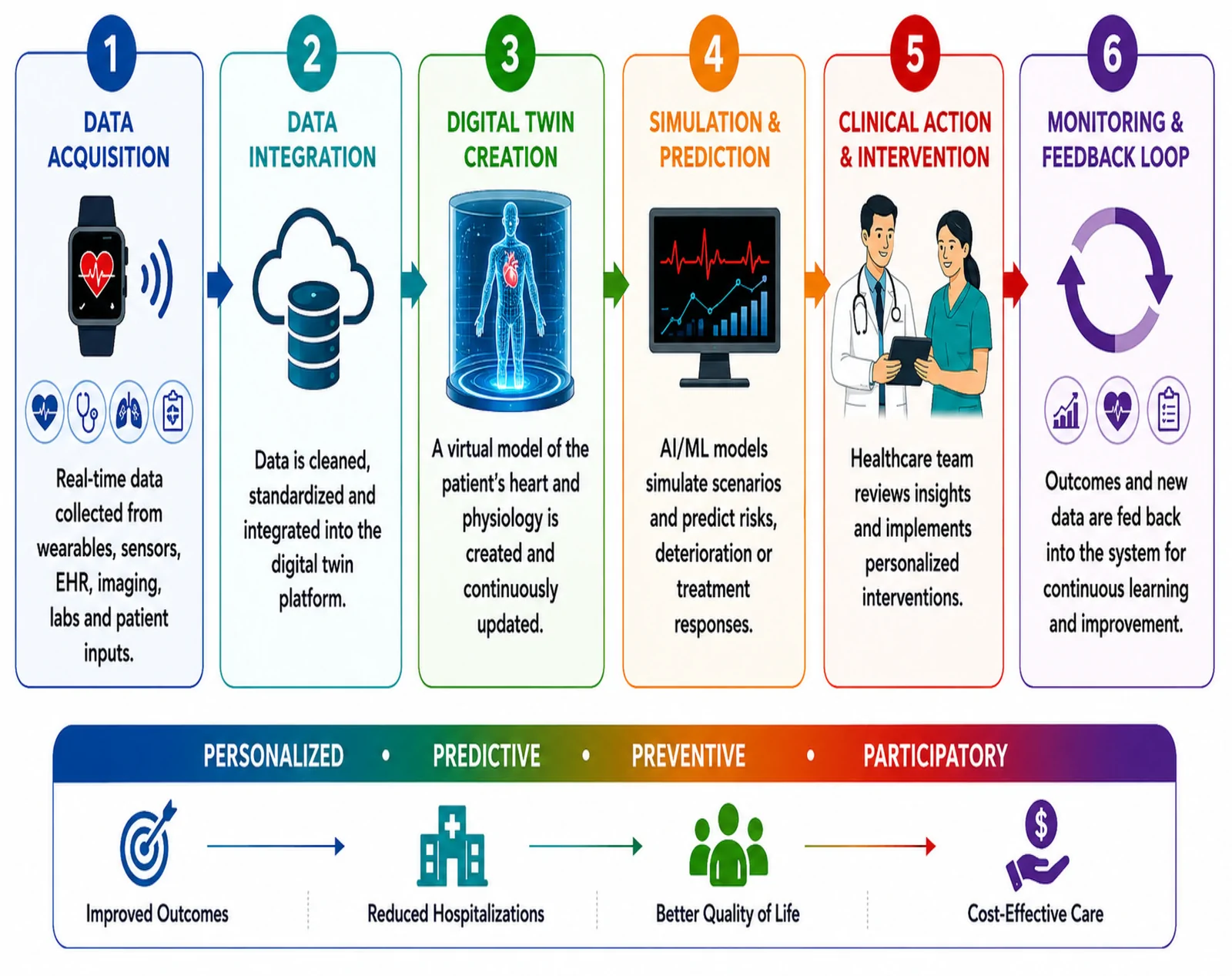
Workflow of digital twin-based cardiac care: conceptual workflow illustrating the integration of patient data, digital twin analytics, clinical decision-making, personalised care, and continuous monitoring. Author-developed conceptual figure based on the synthesis of the reviewed literature; not a validated clinical workflow.

#### 3.4.1 Early detection and risk prediction

Early detection and predictive risk assessment emerged as one of the most extensively reported applications of DTT within cardiovascular healthcare (15, 33). Several studies highlighted that digital twin systems can continuously analyse physiological parameters and identify subtle signs of deterioration before overt clinical symptoms become apparent (23, 26). Heart failure deterioration prediction was frequently discussed because of the chronic and progressive nature of heart failure and its association with recurrent hospital admissions (23, 36). Studies reported that wearable biosensors, remote monitoring devices, and AI-supported predictive analytics could identify changes in heart rate variability, oxygen saturation, blood pressure, respiratory status, and fluid balance, thereby facilitating earlier intervention and reducing avoidable hospitalisation (23, 25). Arrhythmia prediction represented another important application area identified within the literature (9, 34). Computational cardiac models and electrophysiological simulations were used to predict atrial fibrillation recurrence, ventricular tachycardia, and other rhythm abnormalities through analysis of imaging data and real-time electrocardiographic signals (30, 33). Several studies suggested that virtual heart models may improve procedural planning and risk stratification in patients with complex electrophysiological disorders (34, 35). The reviewed studies also highlighted the role of DTT in sudden cardiac event prediction (8, 39). AI-assisted algorithms and computational simulations were reported to improve identification of patients at risk of sudden cardiac death by integrating multiple physiological and imaging parameters into predictive models (33, 39).

#### 3.4.2 Personalised treatment planning

Personalised treatment planning emerged as another major application of DTT in cardiovascular care (11, 15). Several studies described how patient-specific virtual models could simulate individual responses to medications, interventions, and rehabilitation programmes before implementation in clinical practice (13, 41). Medication optimisation was frequently discussed within the context of precision cardiology and AI-supported therapeutic decision-making (8, 11). Digital twin systems were reported to assist clinicians in selecting more personalised pharmacological strategies by predicting haemodynamic responses, identifying potential adverse effects, and analysing patient-specific physiological data (36, 41). Several studies also explored prediction of treatment responses using computational modelling and electrophysiological simulations (30, 33). These approaches were particularly relevant for arrhythmia management, catheter ablation procedures, and structural heart interventions, where digital simulations helped optimise therapeutic planning and procedural precision (34, 35). Tailored rehabilitation planning was another important application reported within the literature (32, 37). Wearable monitoring systems and digital rehabilitation platforms enabled healthcare professionals to individualise exercise programmes according to patients' cardiovascular tolerance, physiological responses, and recovery progress (32). Studies suggested that continuous physiological monitoring improved adherence, patient engagement, and long-term rehabilitation outcomes (37).

#### 3.4.3 Remote cardiac monitoring

Remote cardiac monitoring represented one of the most clinically applicable uses of DTT identified in the review (24, 25). Several studies reported increasing integration of telecardiology systems, wearable biosensors, mobile applications, and Internet of Medical Things (IoMT) technologies into cardiovascular care pathways (27, 40). Telecardiology integration enabled healthcare professionals to remotely monitor patients with chronic cardiovascular diseases and identify early signs of clinical deterioration (24, 27). Home-based monitoring systems facilitated continuous assessment of ECG recordings, oxygen saturation, blood pressure, and activity levels, thereby improving continuity of care and reducing unnecessary hospital visits (23, 25). Several studies highlighted the importance of continuous physiological surveillance for patients with heart failure and arrhythmias (23, 26). Real-time data transmission allowed clinicians and nurses to respond rapidly to abnormal physiological changes and optimise patient management remotely (27, 40).

#### 3.4.4 Critical care and intensive care applications

The reviewed studies demonstrated increasing interest in the application of DTT within cardiovascular intensive care and critical care environments (26, 28). Smart ICU systems integrating predictive analytics, AI-supported alerts, and haemodynamic simulations were identified as emerging innovations in critical cardiovascular care (26). Haemodynamic simulation was frequently discussed as an important application of computational cardiovascular modelling (36). These systems enabled clinicians to predict cardiovascular instability, assess treatment responses, and optimise haemodynamic management in critically ill patients (26). Several studies additionally highlighted the role of real-time deterioration alerts in improving patient safety and reducing delays in intervention (10, 28). Predictive ICU systems were reported to assist healthcare professionals in identifying arrhythmias, respiratory compromise, and haemodynamic instability before clinical deterioration became severe (26).

#### 3.4.5 Surgical and interventional applications

Virtual cardiac modelling and procedural simulation emerged as important applications of DTT within cardiovascular surgery and interventional cardiology (30, 33). Patient-specific computational models enabled clinicians to visualise cardiac anatomy, simulate interventions, and optimise procedural planning before surgery or catheter-based treatment (34, 35). Several studies suggested that pre-procedural simulation improved procedural precision, reduced operative risk, and enhanced patient safety (30, 37). Virtual electrophysiological simulations were particularly useful in arrhythmia management and ablation planning (33).

#### 3.4.6 Cardiac rehabilitation applications

Cardiac rehabilitation applications were increasingly discussed within the reviewed studies (32, 37). DTT-supported rehabilitation systems facilitated individualised exercise planning based on real-time physiological monitoring and patient-specific cardiovascular responses (32). Wearable monitoring technologies enabled clinicians and nurses to monitor adherence, recovery progression, and rehabilitation outcomes continuously (37, 40). Several studies reported that personalised digital rehabilitation programmes improved patient engagement and long-term self-management behaviours (32).

#### 3.4.7 Population health management

Population-level cardiovascular management emerged as an evolving application of DTT within preventive cardiology and public health planning (29, 38). Federated learning systems and large-scale predictive analytics were used to analyse cardiovascular trends, identify high-risk populations, and support preventive healthcare strategies (38). Predictive epidemiology models were reported to improve resource allocation, public health planning, and cardiovascular risk stratification across communities (29). Several studies suggested that population digital twins may contribute to future preventive healthcare systems by enabling earlier identification of vulnerable populations and targeted interventions (11, 38).

### 3.5 Implications of digital twin technology for cardiovascular nursing practice

The reviewed studies demonstrated that DTT may substantially transform cardiovascular nursing practice by supporting predictive monitoring, telehealth integration, data-driven care, and intelligent clinical decision-making (10, 24, 27). The expanding role of cardiovascular nurses within digital twin ecosystems is presented in Figure 4**.**

**Figure 4:**
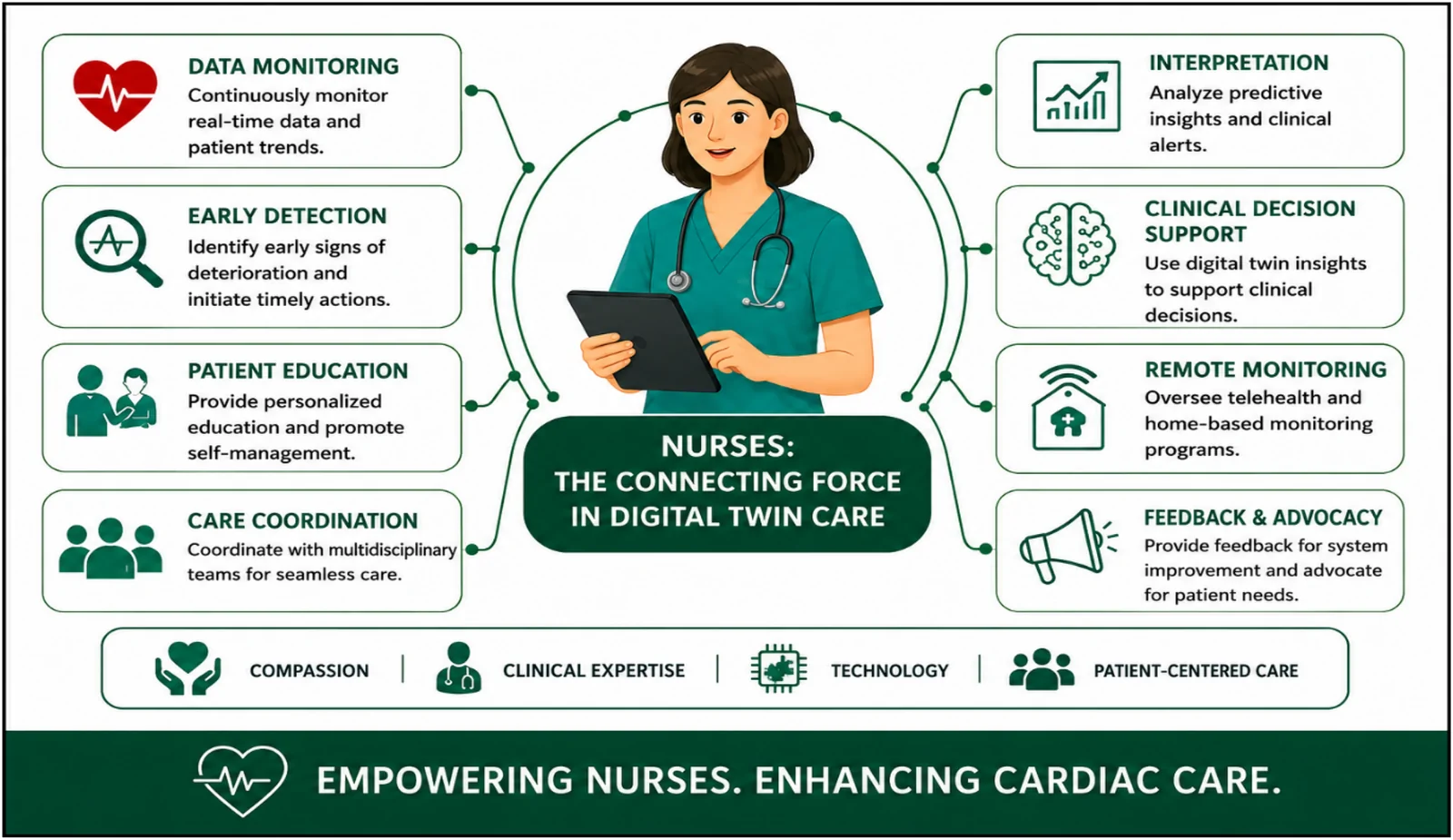
Role of cardiovascular nurses in digital twin-enabled care. Author-developed conceptual figure based on the synthesis of the reviewed literature; represents potential and emerging nursing roles rather than established clinical practice.

#### 3.5.1 Transformation of cardiovascular nursing roles

Several studies highlighted a transition from traditional reactive nursing care towards predictive and preventive cardiovascular nursing practice (10, 11). Nurses were increasingly expected to interpret digital health data, monitor predictive alerts, and participate in AI-assisted patient management (28).

Data-driven nursing practice was identified as a major component of future cardiovascular care models (29, 31). Nurses were required to integrate digital information from wearable devices, telehealth systems, and predictive monitoring platforms into routine patient care and clinical prioritisation (26).

#### 3.5.2 Nursing informatics and digital competency

Digital competency and nursing informatics emerged as important themes throughout the included studies (24, 31). Several authors emphasised the need for nurses to develop competencies in AI interpretation, telehealth systems, wearable technologies, and digital health analytics (10, 29).

The reviewed studies also highlighted the importance of integrating digital health education into undergraduate nursing curricula and continuing professional education programmes (11, 28).

#### 3.5.3 Enhanced clinical decision-making

Digital twin systems were consistently reported to enhance clinical decision-making through predictive analytics and intelligent monitoring systems (8, 28). Early intervention strategies were facilitated through real-time physiological surveillance and automated clinical alerts (26).

Several studies suggested that predictive monitoring systems improved clinical prioritisation, especially in critical care settings where rapid deterioration required prompt nursing intervention (23, 26).

#### 3.5.4 Nurse-led remote monitoring and telehealth

Nurse-led remote monitoring emerged as an important area of cardiovascular digital care (24, 27). Telehealth integration enabled nurses to conduct virtual follow-up, monitor physiological parameters remotely, and coordinate chronic disease management (25).

Virtual nursing models were considered particularly valuable for patients living in rural and underserved regions with limited access to specialist cardiovascular services (24, 27).

#### 3.5.5 Patient education and counselling

Patient education remained a central nursing responsibility within technology-assisted cardiovascular care (29). Nurses played a key role in improving patient understanding of wearable devices, telehealth systems, and digital rehabilitation programmes (32).

Several studies reported that digital health coaching improved patient engagement, medication adherence, lifestyle modification, and self-management behaviours (23, 40).

#### 3.5.6 Impact on patient safety and quality of care

The reviewed literature suggested that DTT may improve patient safety by facilitating earlier recognition of clinical deterioration and reducing delays in intervention (26, 28). Predictive monitoring systems were associated with improved continuity of care, enhanced multidisciplinary collaboration, and reduced adverse cardiovascular events (23).

Several studies also suggested that personalised digital monitoring may contribute to reduced hospital admissions, improved rehabilitation adherence, and more efficient healthcare delivery (27, 32).

### 3.6 Benefits and challenges associated with digital twin technology in cardiovascular healthcare

The reviewed studies identified several important benefits and challenges associated with the integration of Digital Twin Technology (DTT) into cardiovascular healthcare systems (10, 11, 15). Although DTT has shown considerable potential for improving predictive monitoring, personalised treatment, and intelligent clinical decision-making, the literature also highlighted multiple ethical, technological, organisational, and workforce-related barriers that may affect successful implementation (13, 28).

#### 3.6.1 Reported benefits of digital twin technology

One of the most frequently reported benefits of DTT was improvement in continuous patient monitoring and early identification of clinical deterioration (23, 25). Several studies suggested that wearable biosensors, remote monitoring systems, and AI-assisted analytics enabled real-time assessment of physiological changes, thereby supporting timely intervention and reducing delays in care (27, 40). Continuous monitoring was considered particularly beneficial for patients with chronic cardiovascular diseases such as heart failure and arrhythmias, where early detection of worsening symptoms may prevent severe complications and hospital readmissions (23, 26).

Predictive and personalised care represented another major advantage identified across the reviewed studies (11, 15). Patient-specific digital twins were reported to improve risk stratification, simulate treatment responses, and support precision medicine approaches tailored to individual physiological characteristics (13, 41). Computational cardiovascular models enabled clinicians to predict haemodynamic responses, evaluate arrhythmia risks, and optimise intervention planning before treatment implementation (33, 34). Several studies suggested that these predictive capabilities may improve patient outcomes and support more individualised cardiovascular care pathways (36, 39).

Enhanced healthcare efficiency was also widely discussed within the literature (12, 28). Automated monitoring systems and predictive analytics were reported to reduce unnecessary hospital visits, streamline clinical workflows, and improve prioritisation of high-risk patients (10, 26). Smart ICU systems integrating real-time alerts and computational simulations were considered valuable for improving clinical responsiveness and reducing workload associated with manual surveillance (26). In addition, remote monitoring and telecardiology services facilitated continuity of care while reducing pressure on healthcare facilities and outpatient services (24, 27).

Reduced hospitalisation rates were another important benefit identified in several studies (23, 32). Early detection of deterioration through remote physiological monitoring enabled earlier intervention and prevented avoidable emergency admissions (23). Digital rehabilitation systems and telemonitoring programmes also supported long-term self-management, improved adherence to treatment, and reduced recurrent hospitalisation among patients with chronic cardiovascular diseases (32, 40).

#### 3.6.2 Ethical and legal challenges

Despite these benefits, the reviewed studies consistently highlighted significant ethical and legal concerns associated with DTT implementation (14, 28). Data privacy and confidentiality emerged as one of the most important ethical challenges because digital twin systems require continuous collection, integration, and storage of large volumes of sensitive patient information (10, 12). Several authors expressed concern regarding potential misuse of personal health data, unauthorised access, and breaches of patient confidentiality within interconnected digital ecosystems (14, 27). Informed consent and data ownership were also frequently discussed within the literature (11, 14). Questions were raised regarding who owns continuously generated physiological data and how patients can maintain control over the use of their digital health information (12). Some studies suggested that patients may not fully understand the complexity of AI-driven predictive systems and the extent to which their data are used for modelling, simulation, and machine learning processes (29). Algorithm bias represented another important ethical concern identified within the review (28, 39). AI systems trained on limited or non-diverse datasets may generate biased predictions and contribute to inequitable healthcare outcomes, particularly among underrepresented populations (11, 38). Several studies emphasised the importance of developing inclusive datasets and transparent AI governance frameworks to minimise discrimination and ensure equitable cardiovascular care (10).

#### 3.6.3 Technological and organisational challenges

Technological and organisational barriers were consistently identified throughout the reviewed studies (12, 13). High infrastructure and implementation costs were among the most commonly reported challenges because digital twin ecosystems require advanced computational systems, wearable devices, secure cloud infrastructure, and continuous data integration platforms (26, 28). Several authors suggested that the financial burden associated with implementation may limit adoption within resource-constrained healthcare systems (29). Interoperability issues also represented a major challenge (12, 25). Many healthcare institutions use different electronic health record systems, monitoring devices, and software platforms that may not communicate effectively with each other (11). Lack of standardisation was considered a significant obstacle to seamless integration of digital twin technologies into routine cardiovascular care pathways (13). Cybersecurity risks were another major concern identified across the literature (14, 28). Continuous data exchange between wearable devices, cloud platforms, and hospital information systems increases vulnerability to cyberattacks, ransomware, and unauthorised access to sensitive patient information (12). Several studies emphasised the need for robust cybersecurity frameworks and secure digital governance systems before widespread clinical implementation can occur (10).

#### 3.6.4 Workforce and educational challenges

Workforce preparedness emerged as an important challenge related to the future integration of DTT into cardiovascular care (24, 31). Several studies highlighted that many healthcare professionals, including nurses, may lack sufficient training in digital health technologies, AI systems, predictive analytics, and telehealth platforms (10, 29). Limited digital competency among healthcare professionals was repeatedly identified within the literature (11, 28). Nurses are increasingly expected to interpret digital health data, manage wearable technologies, and participate in technology-assisted clinical decision-making; however, formal education and training programmes often remain inadequate (31). Several studies recommended incorporation of digital health competencies into undergraduate nursing curricula, postgraduate education, and continuing professional development programmes (29). Resistance to technological adoption was another challenge identified across multiple studies (24, 27). Some healthcare professionals expressed concerns regarding increased workload, reduced human interaction, dependence on automated systems, and fear of technological replacement (26). Patients may also experience anxiety, distrust, or difficulty adapting to technology-driven healthcare systems, particularly among older adults with limited digital literacy (40).

#### 3.6.5 Challenges in low- and middle-income countries

The reviewed studies highlighted important disparities in digital healthcare implementation between high-income countries and low- and middle-income countries (LMICs) (10, 29). Limited digital infrastructure was identified as a major barrier preventing widespread adoption of DTT in resource-limited settings (11). Inadequate internet connectivity, insufficient access to advanced healthcare technologies, and lack of specialised computational resources were frequently reported challenges (12). Accessibility and affordability barriers were also significant concerns (29). Many healthcare facilities in LMICs may lack the financial capacity to implement advanced AI systems, wearable monitoring technologies, and cloud-based digital ecosystems (13). Several studies suggested that unequal technological access may further widen healthcare disparities and limit equitable delivery of precision cardiovascular care (38).

Beyond technological and infrastructural barriers, implementation of DTT raises important regulatory, reimbursement, explainability, and equity considerations. Clear regulatory and governance frameworks are required to address data privacy, cybersecurity, accountability, clinical validation, and oversight of AI-supported decisions (10, 14, 28). Reimbursement mechanisms and implementation costs may also influence the sustainability and scalability of DTT-enabled monitoring and remote care (12, 29). Furthermore, AI explainability and digital competency are important to enable nurses and other clinicians to appropriately interpret and critically evaluate AI-supported predictions and recommendations (10, 28). Digital equity must also be considered, as unequal access to digital technologies, connectivity, and digital literacy may limit the benefits of DTT among underserved populations and resource-constrained settings (11, 29, 38).

### 3.7 Research gaps and future directions

The reviewed studies identified several important gaps in the current literature and highlighted multiple areas requiring future investigation (11, 28).

#### 3.7.1 Limited nursing-focused evidence

One of the most important gaps identified in the review was the limited availability of nursing-focused research related to DTT in cardiovascular healthcare (24, 27). Although several studies discussed telehealth integration and remote monitoring, very few directly explored the role of cardiovascular nurses in digital twin implementation, workflow integration, or predictive nursing care models (10). Therefore, the nursing implications identified in this review should be interpreted as emerging applications informed by the available evidence rather than as established nursing interventions. The proposed digital twin-integrated cardiovascular nursing care framework is presented in Figure 5.

**Figure 5:**
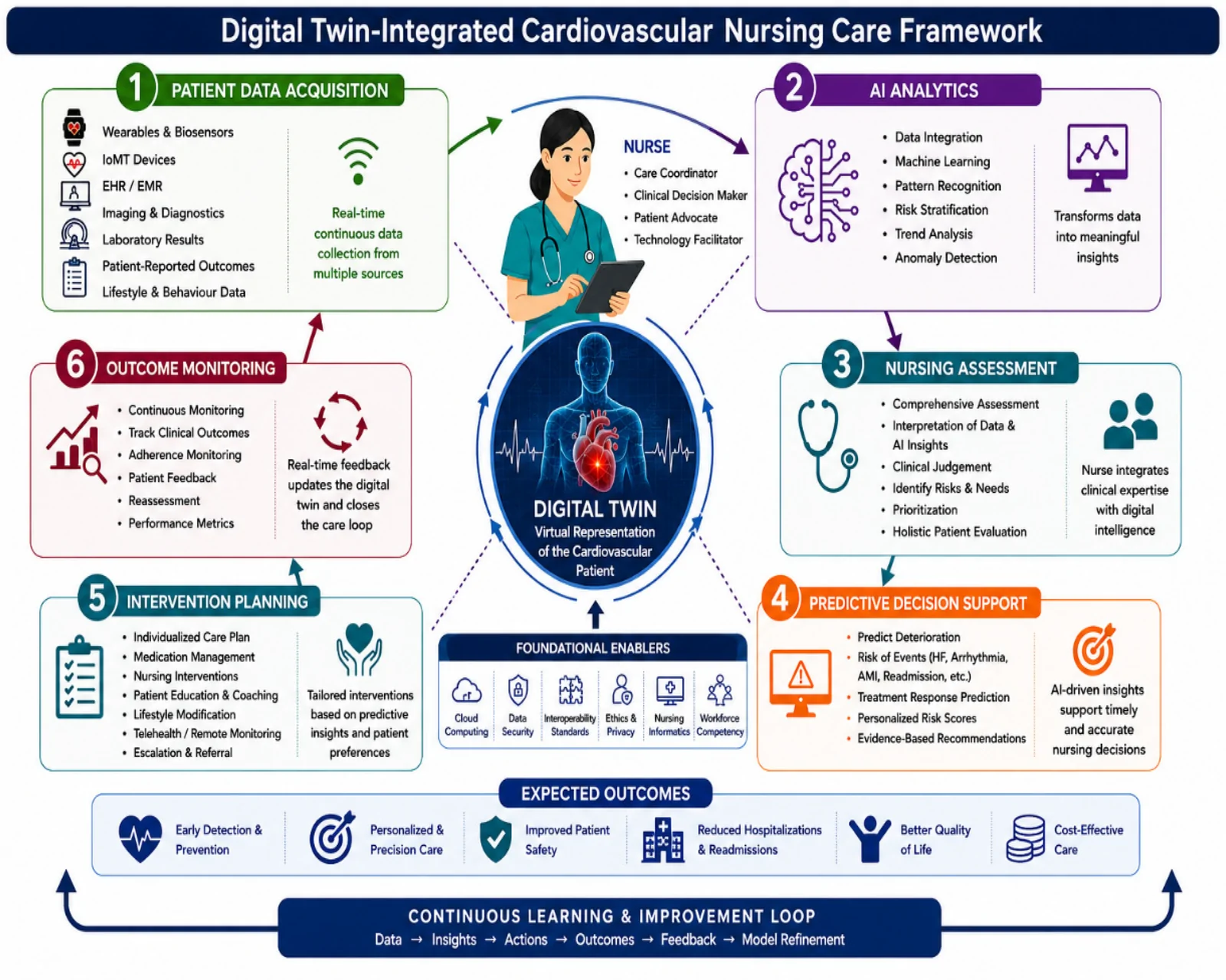
Digital twin-integrated cardiovascular nursing care framework. Author-developed proposed model for integrating digital twin technology into cardiovascular nursing practice (not a validated clinical framework).

#### 3.7.2 Lack of longitudinal and clinical outcome studies

Most included studies were conceptual reviews, modelling studies, or theoretical discussions rather than real-world clinical evaluations (13, 15). There remains a lack of longitudinal clinical trials examining patient outcomes, long-term effectiveness, safety, and sustainability of cardiovascular digital twin systems in routine practice (26).

#### 3.7.3 Need for cost-effectiveness evaluations

Very limited evidence was identified regarding cost-effectiveness and economic sustainability of DTT implementation (12, 29). Future studies are needed to evaluate whether digital twin systems can reduce healthcare expenditure, improve resource utilisation, and justify infrastructure investment within diverse healthcare settings (11).

#### 3.7.4 Necessity for ethical and regulatory frameworks

Several studies emphasised the urgent need for standardised ethical and regulatory frameworks governing AI-assisted cardiovascular care and digital twin systems (14, 28). Clear guidelines regarding data governance, patient consent, algorithm transparency, and accountability remain insufficiently developed (10).

#### 3.7.5 Digital competency and educational gaps

The literature highlighted insufficient preparation of healthcare professionals for future digital healthcare environments (31). There is a need for competency frameworks addressing digital literacy, AI interpretation, telehealth systems, and predictive analytics for cardiovascular nurses and multidisciplinary healthcare teams (29).

#### 3.7.6 Patient acceptance and usability concerns

Patient acceptance of DTT and wearable technologies remains underexplored within current research (27, 40). Future investigations should evaluate patient perceptions, usability, trust, adherence, and psychological responses associated with continuous digital monitoring systems (32).

#### 3.7.7 Applicability in rural and resource-limited settings

Limited evidence was identified regarding implementation of DTT in rural healthcare systems and resource-constrained environments (29). Future research should focus on developing affordable, scalable, and context-specific digital cardiovascular care models suitable for low-resource healthcare settings (11). The proposed future smart cardiovascular nursing care model is illustrated in Figure 6.

**Figure 6:**
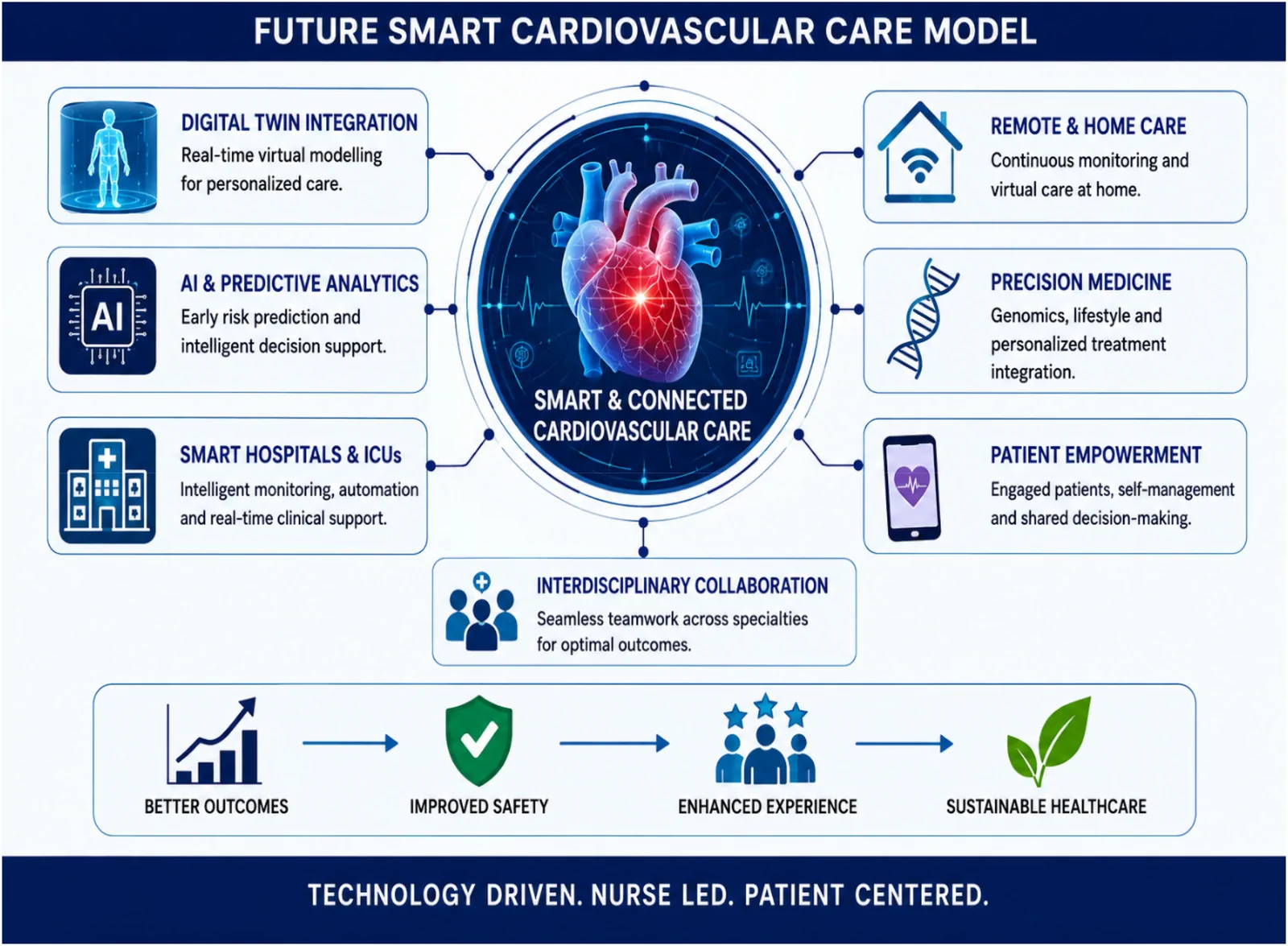
Future smart cardiovascular nursing care model. Author-developed conceptual model based on the synthesis of the reviewed literature; represents a proposed future framework and is not a validated clinical model.

Future studies should directly evaluate nurse-led applications of DTT, including nursing workflow integration, clinical decision-making, patient education, remote monitoring, and nurse-specific implementation models, before these potential roles can be translated into routine practice.

## 4 Strengths and limitations of the review

This scoping review provides a comprehensive and contemporary synthesis of the available evidence regarding Digital Twin Technology (DTT) in cardiovascular healthcare. A major strength of this review is its systematic and rigorous methodological approach based on the Arksey and O'Malley framework and PRISMA-ScR guidelines, which enhanced transparency, reproducibility, and methodological robustness throughout the review process (20, 22). In addition, the review specifically focused on recently published studies between 2020 and 2026, thereby ensuring inclusion of the most current developments in artificial intelligence, computational cardiology, wearable technologies, and digital healthcare ecosystems.

Another important strength of this review is its strong nursing perspective, which addresses a notable gap within the existing literature. Unlike previous reviews that primarily focused on engineering or computational aspects of digital twins, the present review critically examined the implications of DTT for cardiovascular nursing roles, telehealth integration, predictive monitoring, patient education, and digital competency development. The review also integrated findings related to ethical, organisational, educational, and implementation challenges, thereby providing a holistic understanding of the opportunities and barriers associated with DTT integration into cardiovascular healthcare systems.

Despite these strengths, several limitations should be acknowledged. First, the review included only English-language publications, which may have excluded relevant studies published in other languages. Second, because DTT remains an emerging field, many included studies were conceptual reviews, modelling studies, or theoretical discussions rather than real-world clinical implementation studies. Consequently, the available evidence regarding long-term clinical effectiveness, patient outcomes, and cost-effectiveness remains limited. Furthermore, formal methodological quality assessment was not performed because of the heterogeneity of study designs and publication types included in the scoping review. Consequently, the findings should be interpreted as a mapping and synthesis of the available evidence rather than as an assessment of the relative methodological strength of individual studies. Third, considerable heterogeneity existed among the included studies in terms of technological approaches, cardiovascular applications, study designs, and outcome measures, which limited direct comparison of findings. In addition, relatively few studies specifically focused on nursing practice, resulting in limited nursing-specific evidence related to implementation frameworks, workflow integration, and nurse-led digital twin interventions.

Furthermore, most included studies originated from high-income countries with advanced digital healthcare infrastructure, thereby limiting the generalisability of findings to low- and middle-income countries and resource-constrained healthcare settings. Finally, due to the rapid evolution of digital health technologies and artificial intelligence systems, some technological developments may have emerged after completion of the review process. Nevertheless, despite these limitations, the review provides an important and timely synthesis of current evidence and identifies critical directions for future research, policy development, nursing education, and clinical implementation of Digital Twin Technology in cardiovascular healthcare.

## 5 Conclusion

Digital Twin Technology (DTT) is rapidly emerging as a potentially transformative innovation in cardiovascular healthcare, with the potential to influence the future of precision cardiology, patient monitoring, and intelligent clinical decision-making. The findings of this scoping review indicate that DTT is increasingly being explored in combination with artificial intelligence, computational cardiac modelling, wearable biosensors, telecardiology, and predictive analytics to support earlier detection of clinical deterioration, personalised treatment planning, remote monitoring, and continuity of cardiovascular care. The review further highlights that cardiovascular care may be particularly well suited for digital twin integration because of its dependence on continuous physiological monitoring and long-term disease management. Importantly, the reviewed evidence suggests the potential for DTT to support a shift from reactive healthcare towards predictive, preventive, and patient-centred cardiovascular care; however, current clinical evidence remains limited and these potential benefits require further validation in real-world clinical settings.

At the same time, this review identified several critical challenges and research gaps that must be addressed before widespread clinical implementation can be considered. Concerns related to data privacy, cybersecurity, algorithm bias, interoperability, infrastructure costs, and workforce preparedness remain substantial barriers. The review also revealed a significant lack of nursing-focused evidence, particularly regarding nurse-led implementation models, digital competency frameworks, workflow integration, and long-term patient outcomes. Future research should therefore prioritise longitudinal clinical studies to establish effectiveness and patient outcomes, alongside ethical and regulatory framework development, cost-effectiveness evaluations, and nursing-led implementation research to support safe, equitable, and evidence-based integration of DTT into cardiovascular healthcare systems. Meaningful collaboration among clinicians, nurses, engineers, researchers, policymakers, and healthcare organisations will be essential to determine whether the promise of DTT can translate into sustainable improvements in cardiovascular patient care and health outcomes.
